# miR-125a-5p inhibits glycolysis by targeting hexokinase-II to improve pulmonary arterial hypertension

**DOI:** 10.18632/aging.103163

**Published:** 2020-05-19

**Authors:** Li Luo, Lusheng Xiao, Guili Lian, Huajun Wang, Liangdi Xie

**Affiliations:** 1 Department of Geriatrics, The First Affiliated Hospital of Fujian Medical University, Fuzhou, China; 2 Department of General Medicine, The First Affiliated Hospital of Fujian Medical University, Fuzhou, China; 3 Fujian Hypertension Research Institute, The First Affiliated Hospital of Fujian Medical University, Fuzhou, China; 4 Fujian Medical University, Fuzhou, China

**Keywords:** miR-125a-5p, glycolysis, hexokinase-II, pulmonary arterial hypertension

## Abstract

Purpose: The aim of this study was to investigate the effect of microRNAs on the proliferation of pulmonary arterial smooth muscle cells (PASMCs) as a result of targeting hexokinase-II (HK-II) and its mechanism of action.

Results: Differences in metabolic patterns were found between the normal group and monocrotaline-induced pulmonary arterial hypertension (MCT-PH) group. miR-125a-5p decreased glycolysis levels of monocrotaline (MCT)-induced PASMCs by targeting HK-II and inhibiting its proliferation. *In vivo* experiments found that miR-125a-5p agomir upregulated HK-II expression in the MCT-PH. Right ventricular hypertrophy was reversed and cardiac function improved as a result of decreased mean pulmonary artery pressure (mPAP).

Conclusion: *In vitro* and *in vivo* experiments both confirmed that miR-125a-5p could inhibit cell glycolysis and PASMC proliferation to improve PAH by targeting HK-II.

Methods: HK-II overexpression was constructed, and differentially expressed microRNAs were screened for using microarrays. Serum metabolites were detected using Nuclear Magnetic Resonance (NMR). Through screening for characteristic metabolites in rat body fluids and by analyzing biological functions, disordered metabolic pathways were identified. Activity of the miR-125a-5p target HK-II was measured using a luciferase reporter assay. Expression of downstream molecules was measured by RT–qPCR and/or western blot. Glucose consumption and lactic acid production were analyzed and used as a reflection of glycolysis.

## INTRODUCTION

Pulmonary arterial hypertension (PH), also known as "cancer of the cardiopulmonary vascular system", is a fatal disease that is difficult to diagnose and treat. It is characterized by a continuous increase in pulmonary vascular resistance caused by different mechanisms and causes, which eventually lead to chronic cardiopulmonary vascular disease, along with increased right ventricular afterload and right heart failure [1, 2]. The median survival time of untreated idiopathic PH is only 2.8 years, while untreated children have been found to have a worse prognosis with a median survival of only 0.8 years [3]. Along with the advancement of treatment methods [4, 5], the survival time of PAH patients has been increasing each year. Even so, the current status of prevention and treatment for PH is still not optimal, with the 5-year mortality rate of PH still being as high as 40% [6]. During the past two decades, revolutionary progress has been made regarding the pathogenesis of PH. It has been discovered that many factors are associated with the occurrence of PH. These factors include, energy metabolism disorders, bone morphogenetic protein type II receptor signaling pathway, elastase activation, potassium channel dysfunction, mitochondrial abnormalities, transient receptor potential calcium channel activation, endothelial dysfunction and pro-inflammatory response [7]. However, PH pathogenesis has not yet been fully elucidated.

It is well known that self-proliferation and cellular energy abnormalities are two important features of tumor cells [8]. Although PH is not a "tumor" in the true sense, it exhibits many basic features of tumor cells [9], such as excessive proliferation and apoptosis resistance of vascular wall cells [10, 11] and abnormalities in cellular energy metabolism [12, 13]. In PH, pulmonary blood vessels show hypertrophy and hyperplasia of one or more types of vascular wall cells and show an increase in extracellular matrix components, resulting in the thickening and remodeling of the pulmonary vascular wall.

Regarding the energy metabolism characteristics of tumor cells, the German Biochemist and Physiologist, Otto Warburg, proposed that even under oxygen-sufficient conditions, tumor cells still prefer glucose metabolism, rather than ATP-efficient mitochondrial oxidative phosphorylation. This is the famous "Warburg effect" [14]. Vascular cells in PH take up energy in a similar way to that of tumor cells by producing energy through glycolysis rather than oxidative phosphorylation [12, 13]. Researchers used fluorinated deoxyglucose positron emission tomography and found that glucose uptake in lung tissue increased in patients with PH, and that the Warburg effect was enhanced [15, 16]. This seemingly uneconomical energy supply after reprogramming is necessary for the abnormally proliferating cells, to both provide rapid energy for cell growth and as a biosynthetic feedstock. This proliferation pattern and metabolic phenotype are similar to that of tumor cells and is known as the tumorigenicity of PH.

In recent years, researchers have begun to realize that this abnormal metabolic pattern in tumors provide enough energy and material. Therefore, it is hoped that potential tumor cell proliferation can be inhibited by blocking the abnormal energy metabolism pathway of tumor cells to treat the tumors. Amann et al. [17] used RNA interference technology to silence glucose transporter-1 gene, which decreased glucose uptake and lactate production, and also decreased the proliferation and adhesion of cells. The study by Hirschhaeuser [18] and other studies have shown that knockdown of dehydropurine-A in the glycolysis pathway can seriously impair the proliferation and tumor invasiveness of human breast cancer cells and lymphoid stem cells. Tumor cells mainly gain energy through glycolysis for their growth, and inhibition of glycolysis can inhibit proliferation and kill the tumor cells [17, 18].

HK-II is an enzyme that regulates energy metabolism and cell proliferation. HK is the first rate-limiting enzyme in glycolysis, which converts glucose to glucose-6-phosphate with the participation of ATP and plays a key role in regulating glycolysis flux. There are four HK isoenzymes found in mammals: HK-I, HK-II, HK-III and HK-IV [19]. Although both HK-I and HK-II contain two equivalent glucokinase domains, only the two domains of HK-II are catalytically active. Studies have shown that compared with normal cells, HK-II activity is significantly increased in rapidly-growing tumor cells, so that the tumor cells can obtain enough carbon in the absence of oxygen [20]. When HK-II is detached from the mitochondrial outer membrane voltage-dependent anion channel (VDAC), the mitochondrial permeability transition pore is open and transmembrane potential is decreased. Apoptosis promoting factors of the membrane gap, such as cytochrome C, apoptosis-inducing factor and Ca2+, are released into the cytoplasm. Cytochrome C forms apoptotic bodies with the apoptosis activating factors, caspase-9 and dATP, which then recruits and activates caspase-3 to trigger caspase cascades, finally leading to apoptosis [21]. Conversely, HK-II levels increase when the Warburg effect occurs, since HK-II binds to VDAC and the mitochondrial permeability transition pores close, which then inhibits the release of apoptotic factors and decreases levels of apoptosis [22]. Therefore, HK-II is a key node that links energy metabolism with apoptosis.

miRNAs are a group of non-coding small RNAs. The role of miRNAs and HK-II in tumors is relatively clear, but it is unclear whether changes in HK-II in PH are related with the action of miRNAs. If so, which specific miRNA expression changes in PH cause the upregulation of HK-II expression in cells? Do miRNAs regulate target genes to degrade mRNA or do they inhibit protein translation? Are these changes sufficient to alter the abnormal metabolic pattern of pulmonary arterial smooth muscle cells (PASMCs) in PH? These mechanisms are not yet fully elucidated. We proposed a preliminary hypothesis that changes in the expression of certain specific miRNAs may mobilize a pivotal hub between cell proliferation and energy metabolism by blocking abnormal glycolysis pathways in PH, which interfered with cellular energy metabolism and promoted apoptosis, indicating that the inhibition of vascular cell proliferation may reverse PH caused by pulmonary artery remodeling.

## RESULTS

### Differences in metabolic patterns between the normal group and MCT-PH group

**Figure 1:**
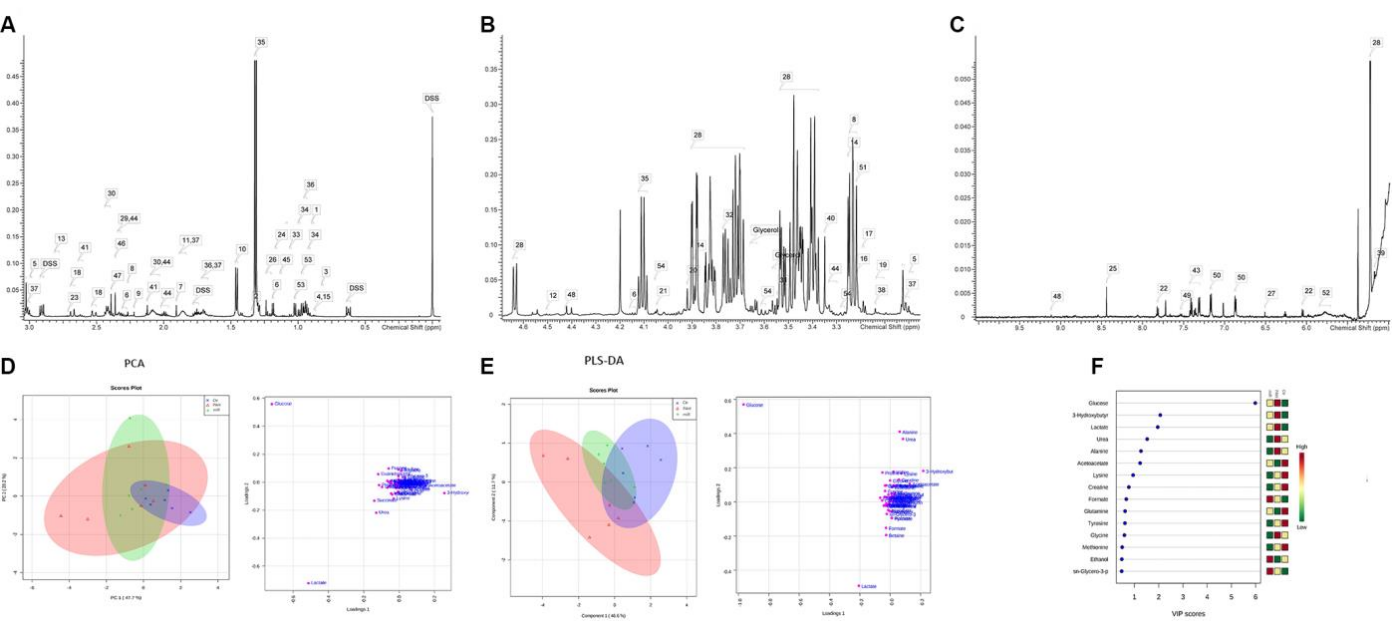
**There are differences in metabolic patterns in the pulmonary hypertension model.** Preparation of rat serum was seen in previous studies [23], and the serum were preserved in -80°C for further metabolomics analysis. Metabolite attribution spectrum of signal fragment of rat arterial blood serum samples: (**A**) attribution range (0.0ppm-3.0ppm interval); (**B**) attribution range (3.0ppm-5.0ppm interval); (**C**) attribution range (5.0ppm-10.0ppm interval). (Associated metabolite number identification: 1: 2-Hydroxybutyrate; 2: 2-Hydroxyisobutyrate; 3; 2-Hydroxyisovalerate; 4: 2-Hydroxyvalerate; 5: 2-Oxoglutarate; 6: 3-Hydroxybutyrate; 7: Acetate; 8: Acetoacetate; 9: Acetone; Alanine; 11: Arginine; 12: Ascorbate; 13: Aspartate; 14: Betaine; 15: Butyrate; 16: Carnitine; 17: Choline; 18: Citrate; 19: Citrulline; 20: Creatine; 21: Creatinine; 23: Dimethylamine; 24: Ethanol; 25: Formate; 26: Fucose; 27: Fumarate); 28: Glucose; 29: Glutamate; 30: Glutamine; Glycerol; 31: Glycine; 32: Guanidoacetate; 33: Isobutyrate; 34: Isoleucine 35: Lactate; 36: Leucine; 37: Lysine; 38: Malonate; 39: Mannose; 40: Methanol; 41: Methionine; 42: ethylsuccinate; 43: Phenylalanine; 44: Proline; 45: Propylene glycol; 46: Pyruvate; 47: Succinate; 48: Trigonelline; 49: Tryptophan; 50: Tyrosine; 51: sn-Glycero-3- phosphocholine; 52: Urea; 53: Valine; 54: myo-Inositol.) Changes of metabolic patterns in rats with pulmonary hypertension: (**D**) PCA analysis, statistical analysis was performed on rat arterial serum samples, NMR spectra were acquired, and samples were analyzed by Chenomx NMR Suite 8.0 to determine the type and concentration of metabolites in the samples. The sample data was normalized using the Pareto Scaling method, and the corresponding score map and load map were obtained by using PCA analysis. (**E**) PLS-DA analysis: In the PLS-DA score map, different colors represent different sample groups. It is found from the figure that the metabolic profile of the NC group samples is distinguishable from the PH group. The load map corresponding to the score map shows that the farther away the metabolites such as Glucose, Lactate, and Alanine are from the center point, the greater the contribution to the distinction between samples. (**F**) VIP is a sort of variable weight importance to provide the most important variables and how important they are in each group. The larger the VIP value, the greater the contribution they make in the differentiation of the sample. Generally, the variable with a default VIP greater than 1 has a significant difference.

The attribution ranges of the signal-dependent fragments of the rat arterial serum samples were 0 ppm-3.0 ppm, 3.0 ppm-5.0 ppm and 5.0 ppm-10.0 ppm (Figure 1A–1C). Nuclear magnetic resonance (NMR) spectra were acquired and the samples were analyzed using Chenomx NMR Suite 8.0 to determine the type and concentration of the metabolites. The sample data was normalized using the Pareto Scaling method, and the corresponding score map and load map were obtained through PCA analysis. In the PLS-DA score map, different colors represent different sample groups. It was found that the metabolic profile of the normal group samples were distinguishable from MCT-PH group. The load map corresponding to the score map showed that the farther away from the center point the metabolite, such as glucose, lactate and alanine, the greater their contribution to the distinction between samples (Figure 1D–1F).

**Figure 2:**
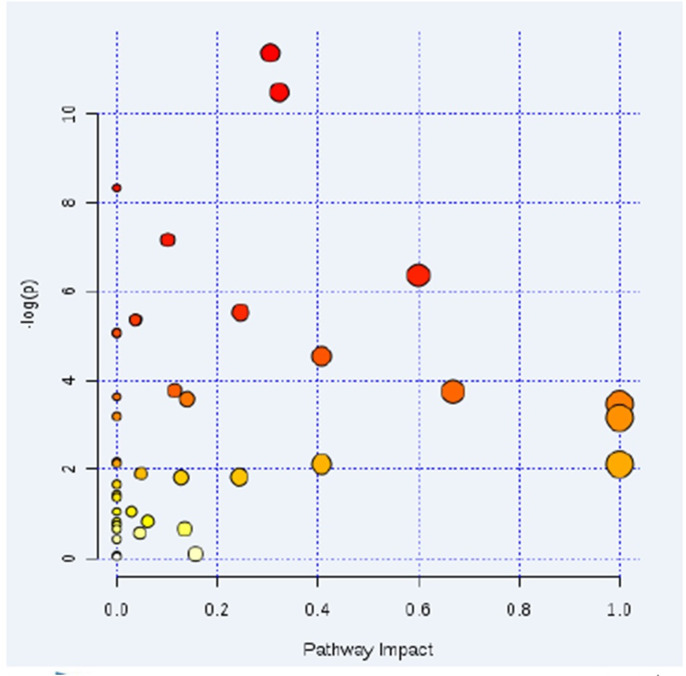
**Pathway analysis found four significant differential metabolic pathways.** Using nuclear magnetic resonance (NMR) metabolomics analysis, the increase of glucose uptake in PH rats, the accumulation of various glycolysis products such as lactic acid, and the activation of glycolytic pathway in PH rats were observed. This data indicated that there was a phenomenon in vascular cell proliferation and energy metabolism in PH rats. (1. Glycolytic pathway, 2. Arginine and proline metabolic pathway, 3. Butyric acid metabolic pathway, 4. Glycine, serine and threonine metabolism.)

Using NMR metabolomics analysis, it was observed that the increase of glucose uptake in MCT-PH rats, the accumulation of various glycolysis products, such as lactic acid, and the activation of the glycolysis pathway, indicate abnormal vascular cell proliferation and energy metabolism in MCT-PH rats (Figure 2).

### mi-RNA is related with increased HK-II expression

**Figure 3:**
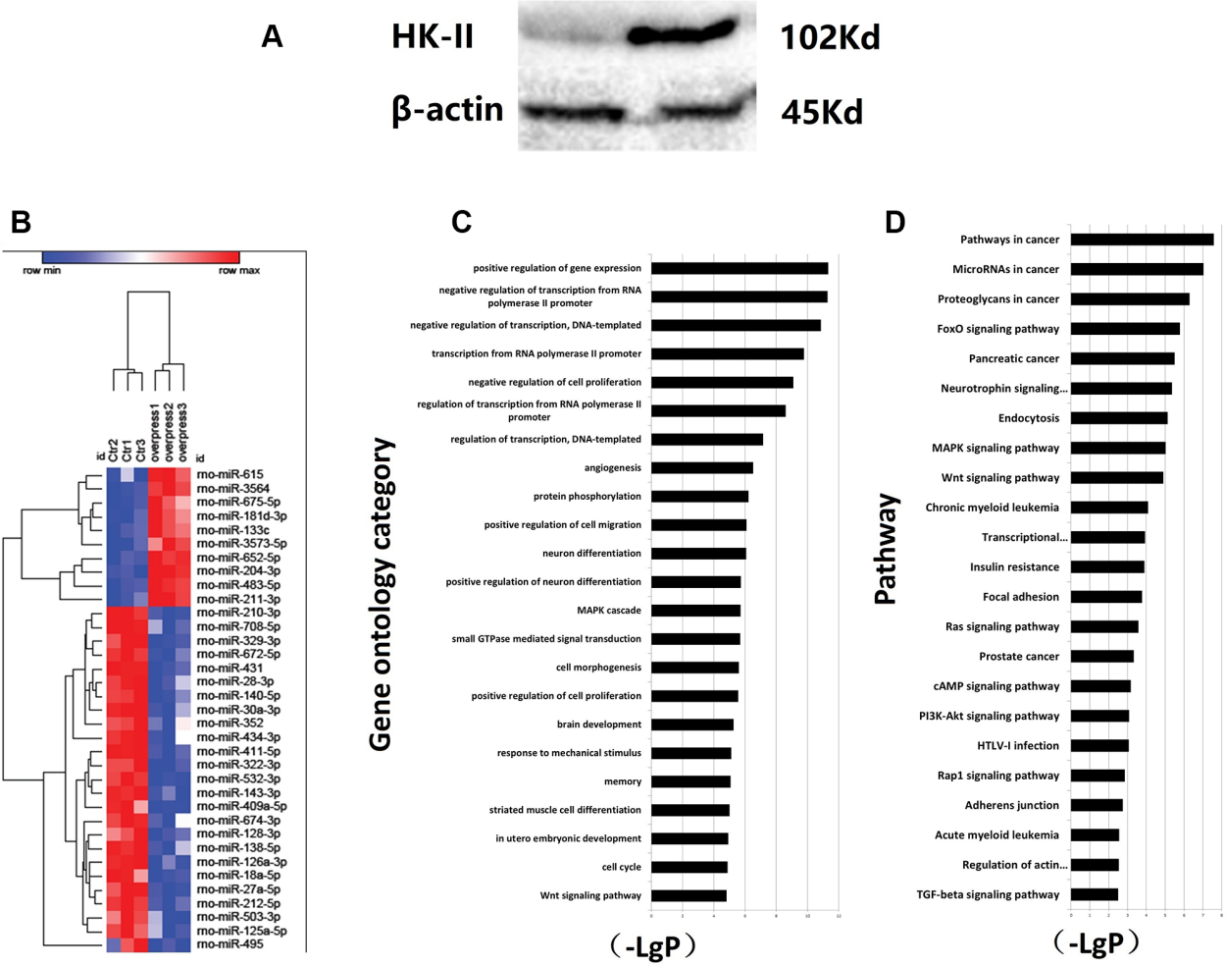
(**A**) Increased expression of HK2 in PH induced by MCT. (**B**) Overexpress HK2 lentivirus and differential MICRORNA screening. PASMCs were transfected in vitro and screened for negatively correlated miRNA molecules with HK-II expression. The control group and PASMCs overexpressing HK2 were detected by miRNA gene expression profiling. (**C**) Regulated gene expression of cell proliferation was significantly active. Sequence-based miRNA target gene prediction method was applied. The number of target genes predicted by this differential miRNA using MIRANDA was 2432. Based on the number of genes contained in differentially significant function and their degree of enrichment in the database, a targeted map can be created for significant function according to enrichment order. In the figure, the ordinate is the function of differential gene, and the abscissa is enrichment. Each column on the graph represents a significant differential gene function. The greater the difference in gene function, the higher the ranking. (**D**) Tumor-associated metabolism was active through expression. Pathway analysis was based on the KEGG database. Fisher's exact test and chi-square test were used for differential genes. Based on the analysis results, significantly up-regulated gene pathway can be used to construct a map of differential gene pathways. The ordinate is the name of differential gene pathway, and the abscissa is negative logarithm of p value (-LgP). The larger the value, the smaller the p value.

According to the results, there was an increase in HK-II expression in MCT-PH (Figure 3A). Clustering analysis was used to detect the miRNAs and samples based on the signal values of each miRNA. In the clustering results of the miRNAs, it was found that miRNAs that are clustered have a similar expression in the samples and may perform similar functions.

In the clustering diagram, abscissa represents the sample name between the groups, while the ordinate represents the differential miRNA. Red indicates that the differentially expressed miRNA has a high expression value in the grouped sample, and green indicates that the differentially expressed miRNA has a low expression value (Figure 3B). Based on the analysis results, each differential gene that was found to be significantly upregulated can be used to construct a profile of significant differential gene function. In the figure, the ordinate is the difference gene function name, and the abscissa is the negative logarithm of p value (-LgP). The larger the value, the higher the functional significance level of the differentially expressed gene. Thus, the results show that gene expression inhibited cell proliferation was significantly active (Figure 3C). GO enrichment analyses were used by DAVID as a comprehensive analysis tool, and 3 types of gene function were classified by biological processes and molecular functions. Pathway analysis was based on the KEGG database. The pathways of target genes were analyzed using Fisher's exact test and chi-square test. A p value of <0.05 was used to screen significant pathways. The results show that tumor-associated metabolism was significantly active (Figure 3D).

### miR-125a-5p decreases glycolysis induced by MCT-induced PASMCs by targeting HK-II and inhibiting its proliferation

**Figure 4:**
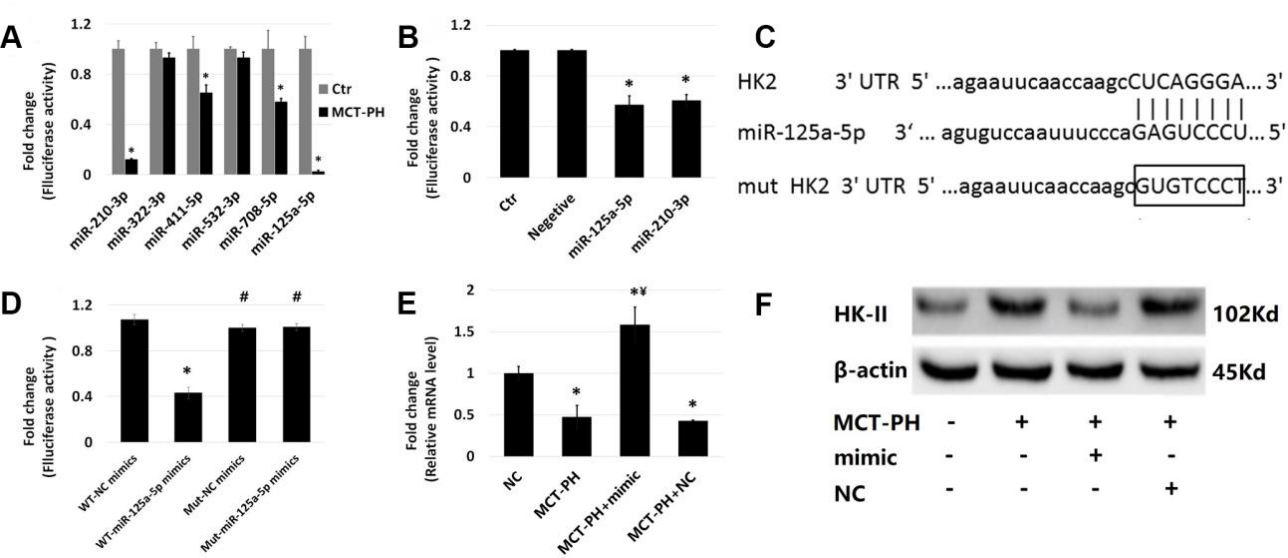
**The expression of each microRNA in primary PASMCs of PH rats induced by MCT was verified by RT-qPCR.** (**A**) RT-qPCR was used to detect the expression of six microRNAs that may act on HK-II in PASMCs of PH: rno-miR-411-5p, rno-miR-210-3p, rno-miR-532-3p, rno-miR-125a-5p, rno-miR-322-3p, rno-miR-708-5p. (**B**) Dual luciferase assay confirmed that miR-210-3p and miR-125a-5p can inhibit luciferase activity. The 3'-UTRs containing normal HK-II mRNA were cloned into the MCS (Multiple cloning site) in the pmirGLO vector to construct the HK2-125/210-pmirGLO vector. This vector was then co-transfected into 293T cells with these two microRNA mimics to determine whether the microRNA acts on the target gene of plasmid by detecting luciferase activity. (**C**) The bioinformatics analysis targeting algorithms (TargetScan and http://microRNA.org) showed that miR-125a-5p contained candidate binding sites in the 3’-UTR of HK-II mRNA. (**D**) The results of luciferase reporter gene assay showed that miR-125a-5p mimic significantly inhibited firefly luciferase activity of wild-type plasmid vector, while firefly luciferase activity of mutant plasmid vector hardly changed. (**E**) Transfection with miR-125a-5p mimic significantly increased the mRNA expression of miR-125a-5p. (**F**) The expression of HK-II protein in PASMCs was significantly increased in PH group, and the expression of HK-II in PH+miR-125a-5p mimic group was significantly decreased. The results demonstrated that miR-125a-5p negatively regulates the expression of HK-II in PASMCs. (*P<0.05, compared with control group, #P<0.05, compared with WT-miR-125a-5p mimics group, ¥P<0.05, compared with PH group).

Due to the increased expression of HK-II in MCT-PH rats, we selected the top 6 miRNAs based on the fold change value of miRNAs whose expression profile chip data was downregulated (miR-411-5p, miR-210-3p, miR-532-3p, miR-125a-5p, miR-322-3p and miR-708-5p). Primer information is shown in Table 1. RT-qPCR was used to detect the expression of the PASMCs. There was no significant difference found between the expression of miR-532-3p and miR-322-3p, while the expression of the other four microRNAs in PASMCs had significantly decreased. miR-125a-5p and miR-210-3p showed the most pronounced decreases (Figure 4A). The bioinformatics analysis targeting algorithms (TargetScan and http://microRNA.org) showed that miR-125a-5p contained candidate binding sites in the 3’-UTR of HK-II mRNA (Figure 4C). Combining these results with gene chip results, we selected miR-125a-5p for further validation.

**Table 1:** Gene and sequence.

**Gene**	**Sequence (5’-3’)**
miR-411-5p	F:CGCGCGCGTAGTAGACCGTATAG
	R:AGTGCAGGGTCCGAGGTATT
	RT:GTCGTATCCAGTGCAGGGTCCGAGGTATTCGCACTGGATACGACCGTACG
miR-210-3p	F:CGCTGTGCGTGTGACAGC
	R:AGTGCAGGGTCCGAGGTATT
	RT:GTCGTATCCAGTGCAGGGTCCGAGGTATTCGCACTGGATACGACTCAGCC
let-532-3p	F:CGCCTCCCACACCCAAGG
	R:AGTGCAGGGTCCGAGGTATT
	RT:GTCGTATCCAGTGCAGGGTCCGAGGTATTCGCACTGGATACGACTGCAAG
miR-322-3p	F:CGCGCGAAACATGAAGCGCT
	R:AGTGCAGGGTCCGAGGTATT
	RT:GTCGTATCCAGTGCAGGGTCCGAGGTATTCGCACTGGATACGACTGTTGC
miR-708-5p	F:CGCGCGAAGGAGCTTACAATCTA
	R:AGTGCAGGGTCCGAGGTATT
	RT:GTCGTATCCAGTGCAGGGTCCGAGGTATTCGCACTGGATACGACCCCAGC
miR-125a-5p	F:CGCGTCCCTGAGACCCTTTAAC
	R:AGTGCAGGGTCCGAGGTATT
	RT:GTCGTATCCAGTGCAGGGTCCGAGGTATTCGC IACTGGATACGACTCACAG
U6	F:CTCGCTTCGGCAGCACATATACT
	R:ACGCTTCACGAATTTGCGTGTC
	RT:ACGCTTCACGAATTTGCGTGTC

In order to identify whether the miR-125a-5p can bind to the 3'-UTRs of HK-II mRNA, 3'-UTRs containing normal HK-II mRNA were cloned into the pmirGLO multiple cloning site (MCS) to construct a HK-II-125 pmirGLO vector. This vector was then co-transfected into 293T cells with microRNA agomir to determine whether the microRNAs act on the target gene of the plasmid by detecting luciferase activity.

miR-125a-5p was selected to further verify if its regulation of HK-II protein is a direct effect. Luciferase reporter gene assay showed that the miR-125a-5p mimic significantly inhibited the firefly luciferase activity of the wild-type plasmid vector, while the mutant plasmid vector showed little change in its firefly luciferase activity (Figure 4D). The above results indicate that miR-125a-5p can directly bind to the 3'-UTRs of HK-II mRNA, suggesting that miR-125a-5p may directly act on HK-II mRNA to regulate protein expression. In order to verify the effect of miR-125a-5p on HK-II, miR-125a-5p was transfected into primary PASMCs and changes in HK-II protein expression were observed. The results of the RT-qPCR showed that miR-125a-5p expression significantly decreased in MCT-PH group, while miR-125a-5p mimic transfection could increase miR-125a-5p levels (Figure 4E). HK-II protein expression in PASMCs significantly increased in MCT-PH group, and the level of HK-II expression in the miR-125a-5p mimic group significantly decreased, which indicates that miR-125a-5p negatively regulated the expression of HK-II in PASMCs (Figure 4F).

**Figure 5:**
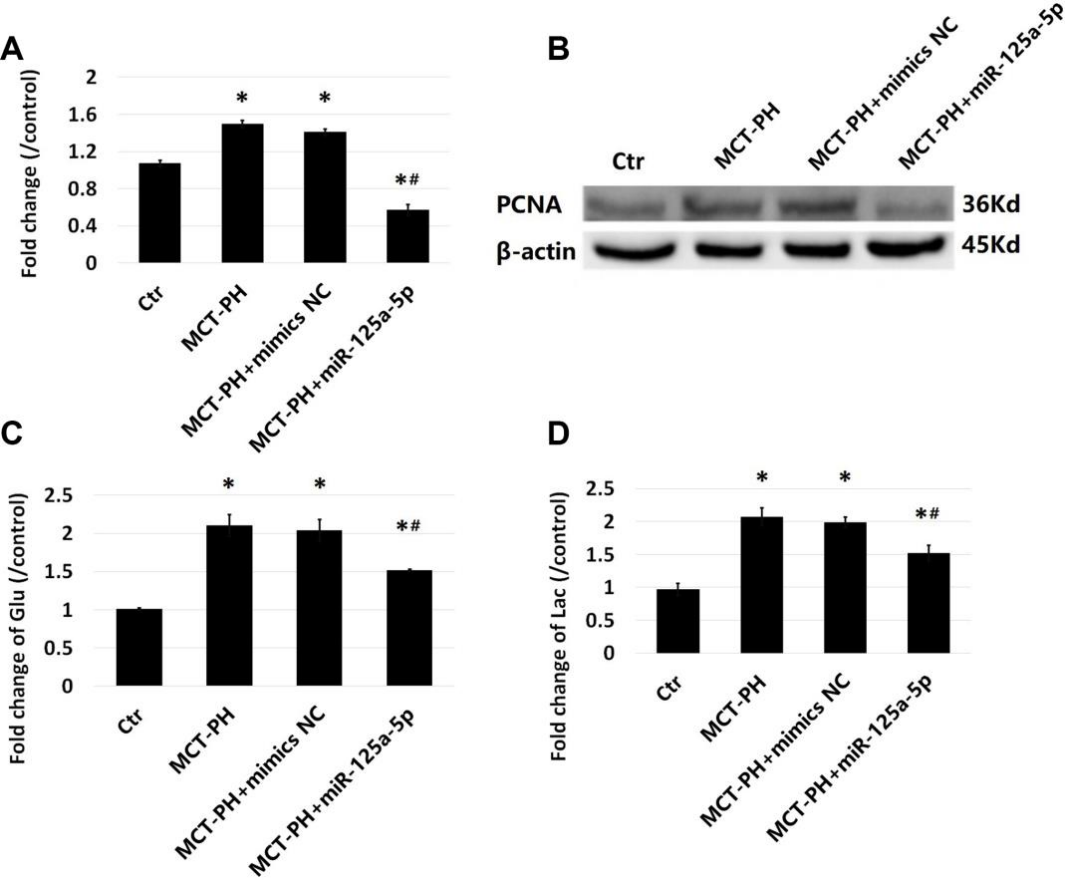
**Effect of miR-125a-5p on PASMCS proliferation.** Cell proliferation levels were determined by CCK-8 kit (**A**) and proliferating cell nuclear antigen (PCNA) (**B**). Glucose consumption (**C**) and lactic acid content (**D**) were measured by assay kits. (*P<0.05, compared with control group, #P<0.05, compared with PH group).

In order to investigate the effect of miR-125a-5p on the proliferation of the PASMCs, miR-125a-5p was transfected into normal and MCT-PH group PASMCs. The results show that the proliferation level of PASMCs in the MCT-PH group was significantly higher than that of the normal group. The proliferation level of PASMCs transfected with the miR-125a-5p mimic was significantly lower than that of the MCT-PH group and MCT-PH + negative control group (Figure 5A and 5B). It was also shown that the transfection of exogenous miR-125a-5p could inhibit PASMC proliferation in MCT-PH.

The glucose consumption of the PASMCs in MCT-PH group was significantly higher than that of the PASMCs in normal group (Figure 5C). The levels of lactic acid produced by PASMCs transfected with miR-125a-5p was significantly higher than that produced under normal conditions (Figure 5D). After miR-125a-5p mimic intervention, glucose consumption and lactic acid production of the PASMCs decreased significantly, suggesting that glycolysis in PASMCs increases under MCT conditions and that miR-125a-5p mimic intervention could decrease glycolysis in PASMCs.

### miR-125a-5p agomir upregulates HK-II expression of PASMCs *in vivo*, which decreases mPAP, leading to the reversal of right ventricular hypertrophy and improvement of cardiac function

**Figure 6:**
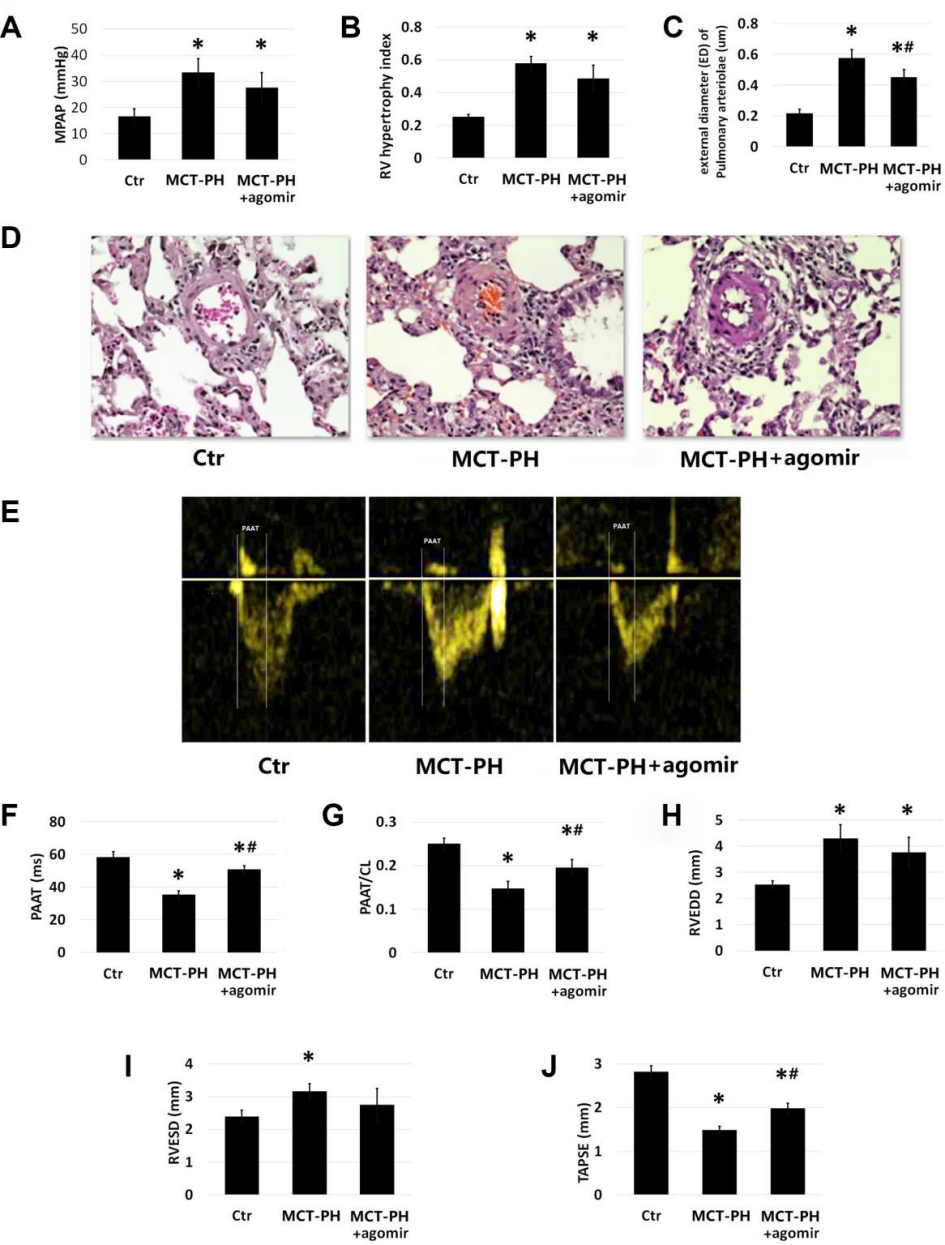
(**A**) The mPAP data were collected and Rated by Powerlab-ML221 (AD Instruments, New South Wales, Australia). (**B**) RVHI was assessed by weighing the RV separately from the left ventricle (LV) with the septal wall (SW). (**C**) Paraffin-embedded sections were prepared and stained with hematoxylin and eosin (H&E). (**D**–**J**)Pulmonary arterioles between 50 and 200 μm in external diameter (ED) were chosen for morphological analysis. Wall thickness (WT) and ED of pulmonary arteries were measured using IPP 6.0 image analysis software (Media Cybernetics). The remodeling of pulmonary arterioles was calculated as WT% = 2 x WT/ED x100%. The probe of color Doppler ultrasound system GE vivid-E9 was placed next to the sternum and measured the pulmonary artery acceleration time (PAAT), right ventricular end thickness (RVWT), right ventricular end systolic diameter (RVESD), right ventricular end diastolic diameter (RVEED), tricuspid annular systolic excursion (TAPSE). The data measured by 3 cardiac cycles is generally taken and averaged. (*P<0.05, compared with control group, #P<0.05, compared with PH group)

The mean pulmonary artery pressure (mPAP), RVHI and pulmonary vascular remodeling values measured in MCT-PH group were significantly higher than those of MCT-PH agomir group, (Figure 6A–6D), while the TAPSE, PAAT and PAAT/CL values of MCT-PH group were significantly lower than those of MCT-PH agomir group (p<0.05). Although the RVEDD and RVESD values of MCT-PH group were higher than that of MCT-PH agomir group, the difference was not significant (Figure 6E–6J).

**Figure 7:**
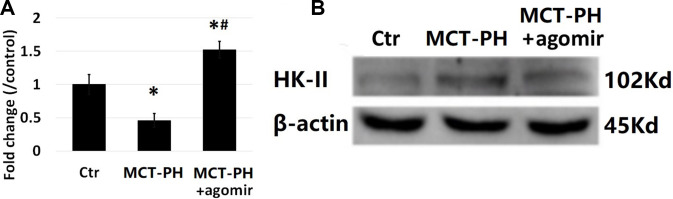
**Detection of pulmonary vascular miR-125a-5p and HK-II expression.** (**A**) RT-PCR assay showed that the expression of miR-125a-5p in PASMCs was significantly lower in PH group. The level of miR-125a-5p expression in PH+ agomir group was higher than that in PH group. (**B**) WB results showed that the expression of HK-II was significantly increased in the PH group, thus demonstrating that miR-125a-5p negatively regulates the expression of HK-II in PASMCs. (*P<0.05, compared with control group, #P<0.05, compared with PH group).

The expression of miR-125a-5p in the PASMCs decreased significantly in MCT-PH group (Figure 7A), while miR-125a-5p expression in MCT-PH agomirgroup was higher than that of MCT-PH group (Figure 7B). The HK-II expression of the PASMCs of MCT-PH group significantly increased compared with that of MCT-PH agomir group, demonstrating that miR-125a-5p negatively regulates the expression of HK-II in PASMCs. These results are consistent with cell levels and results of the *in vivo* experiments.

## DISCUSSION

PH exploration has not slowed down during the past few decades. Along with an increased understanding of PH pathophysiology, effective therapeutic drugs for PH, such as endothelin receptor antagonists, soluble guanylate cyclase, type 5 phosphodiesterase inhibitor and prostaglandins, have made substantial progress in targeting key molecular pathways. However, PH has not been cured until now, and the pace of identifying new and effective treatments needs to be kept going.

There are many cell types involved in PH, such as endothelial cells, fibroblasts, inflammatory cells and abnormal PASMCs [24]. In response to hypoxia, inflammation and other stimuli, pulmonary endothelial cells often form plexiform lesions in PH along with PASMCs, and fibroblasts [25]. Histological examination of lung tissue from patients with idiopathic PH has identified the Infiltration of inflammatory cells, such as lymphocytes, macrophages, dendritic cells and mast cells [24]. Outer membrane fibroblasts respond rapidly to vascular stress, and the proliferation rate of fibroblasts is multiplied especially under hypoxic conditions and MCT [26]. Excessive proliferation of PASMCs and apoptotic resistance are considered as an important pathological basis for PH pathogenesis, while the dynamic balance between proliferation and apoptosis of PASMCs is essential for maintaining normal vascular function. Therefore, this study mainly used PASMCs as targets to study the mechanism of PH pathogenesis.

Among preclinical models of PH, MCT animal models offer the advantage of being able to mimic several key aspects of human PH, including vascular remodeling, proliferation of smooth muscle cells, endothelial dysfunction, upregulation of inflammatory cytokines and right ventricle failure, which requires only a single drug injection [27]. PH is a proliferative arteriopathy associated with a glycolytic shift during heart metabolism. An increase in glycolytic metabolism can be detected in the right ventricle during PH. Expression levels of glycolysis genes in the right ventricle during glycolysis that occur in MCT-induced PH remain unknown [28]. The changes of the metabolic profile during the pathogenesis of MCT-PH mainly involve lipid metabolism, glycolysis, energy metabolism, ketogenesis and methionine metabolism. Metabolic dysfunction is involved in the development and progression of PH [29]. A previous study has demonstrated that EVs isolated from circulation or lungs of mice with MCT-PH cause right ventricular hypertrophy and pulmonary vascular remodeling when injected into healthy mice [30]. Our results are consistent with the results of this study.

Aerobic glycolysis is a phenomenon common in various tumor cells, in which even with sufficient levels of oxygen, cells tend to rely on glycolysis to produce energy needed for cellular activity [4]. MCT was able to induce an increase in glycolysis of PASMCs in this study. Only two ATPs molecules can be produced per glucose molecule by glycolysis, while one molecule of glucose can produce up to 36 ATP molecules through oxidative phosphorylation. This seemingly uneconomical energy supply after reprogramming is necessary for the abnormally proliferating cells. On the one hand, glycolysis can provide more energy. On the other hand, metabolism products, such as glucose-6-phosphoric acid and pyruvic acid, are important sources of fatty acid and nucleotide synthesis. In non-small cell lung cancer, deguelin exposure inhibits glycolysis by inhibiting Akt-mediated HK-II expression, thereby inhibiting cancer cell growth [22]. In hepatoma cells, miR-199a-5p directly targets HK-II in the 3' untranslated region, decreasing glucose consumption and lactic acid production levels, as well as decreased cell 6-phosphate glucose and ATP levels, thereby inhibiting cell proliferation and tumorigenesis [31].

The above results suggest that glycolysis can be effectively decreased and cell proliferation can be inhibited by decreasing HK-II expression. HK-II is an important rate-limiting enzyme for glycolysis. A transcriptome sequencing of tissue samples from 27 different tissues of 95 individuals showed that HK-II is highly expressed only in certain tissues, such as fat, bone marrow, colon and testis [32]. However, in abnormally proliferating cells, such as tumors, it has been found that HK-II expression is abnormally elevated. In this study, we found that HK-II protein and mRNA levels were significantly elevated in MCT-PH rat models. MCT-PH was accompanied by changes in metabolic patterns, including fatty acid beta oxidation and increased glycolysis, reduced aerobic oxidation of sugars and the occurrence of the Warburg effect [33], which may be associated with the abnormal proliferation of PASMCs and vascular remodeling in MCT-PH. Therefore, clarifying the mechanism of glycolysis in MCT-PH may be of great significance for the study of MCT-PH pathogenesis.

As a key enzyme of glycolysis, HK-II can not only can regulate glycolysis, but can also participate in the formation of mitochondrial membrane permeability transition (MMPT) pores in combination with VDAC proteins, which has been shown to effectively inhibit tumor growth by destroying their binding [34, 35]. HK-II can also participate in the regulation of autophagy by binding to the autophagy inhibitor, mTOR complex 1 (TORC1) [36]. Triggers such as glucose and insulin can activate the HK-II promoter, and methylation of the HK-II promoter can also regulate HK-II expression [37]. In addition, HK-II is also regulated by non-coding RNAs, such as microRNAs [11].

Different microRNAs play different roles in PH development. miR-124 can cause abnormal metabolism and proliferation of PH endothelial cells and fibroblasts by regulating polypyrimidine-tract-binding protein (PTPB1) and PKM1/PKM2 [38, 39]. miR-138 and miR-25 downregulate the mitochondrial calcium unidirectional transporter and cAMP response element binding protein 1, which affect mitochondrial dynamics and contribute to cancer-like phenotypes of PH [40]. miR-424 and miR-503 are downregulated in PH, which leads to resistance of cell proliferation in endothelial cells and inhibits PASMC proliferation induced by the conditioned medium [41]. AntagomiR-20a can specifically inhibit miR-20a, which leads to the activation of downstream targets of BMPR2, such as Id-1 and Id-2, and decreased PASMC proliferation [42].

Due to increased expression of HK-II in PH, this study primarily screened microRNAs that acted on HK-II and lowered its expression in MCT-induced PASMC. We predicted that these microRNAs may directly act on HK-II and using the gene chip we identified 6 different microRNAs. The results showed that except for miR-322-3p and miR-532-3p expression, which had no difference on MCT-induced PASMCs, and the other four microRNAs resulted in decreased expression to different extents, with miR-125a-5p causing the most decrease. Widespread, tonic control of mRNAs encoded by genes relevant to blood pressure regulation by endothelial microRNAs [43]. miR-125a-5p ameliorated MCT-PH in rats, has a negative feedback regulation with TGF-β1 and IL-6, and regulates the proliferation and apoptosis of PASMCs by directly targeting STAT3 [44]. The decreased expressions of miR-125a-5p and miR-125b-5p are negatively associated with upregulation of preproET-1 expression in aorta of stroke-prone spontaneously hypertensive rats (SHR-SPs) [45]. We selected miR-210-3p and miR-125a-5p for the dual luciferase reporter gene experiments to verify whether they act on HK-II. The results suggest that these two microRNAs can inhibit the activity of luciferase to different degrees, while miR-125a-5p showed a higher inhibition rate. miR-125a-5p expression in PASMCs was found to be significantly downregulated under MCT-induced conditions, and miR-125a-5p mimic intervention could decrease HK-II expression and glycolysis, while inhibiting cell proliferation.

A decrease in miR-125a-5p expression has been observed in almost all tumors. In recent years, it has been found that miR-125a-5p is differentially expressed in various tumor tissues, such as colon cancer, pancreatic cancer, cervical cancer, breast cancer and gastric cancer, affecting tumor cell proliferation, apoptosis and migration [5–7]. miR-125a-5p can decrease the proliferation of breast cancer cells by interfering with ERBB2 protein expression. miR-125a-5p may provide a new direction in gene therapy for breast cancer [46–50]. Tong et al. reported that miR-125a-5p inhibited cell proliferation and promoted apoptosis by targeting BcL2, BcL2L12 and Mcl1 in colon cancer [51]. The overexpression of miR-125a-5p or knockdown of LIMK1 was found to lead to decreased viability of non-small cell lung cancer tissues and cells, decreased IC50 of cisplatin and downregulated the expression of drug-resistant proteins. In addition, studies have shown that microRNAs, including miR-125b-5p, miR-199a, miR-384-3p and miR-214, can inhibit glycolysis and cell proliferation in PH, hepatocellular carcinoma [52], diabetic retinopathy [53], non-small cell lung cancer [54] and other diseases [55]. This suggests that there may be other microRNAs in the PASMCs that can regulate glycolysis. Therefore, microRNAs that may play a more important role in glycolysis need to be identified and further explored.

Taken together, this experiment demonstrated that HK-II expression increased and miR-125a-5p levels decreased under MCT-PH. *In vitro* and *in vivo* experiments both confirmed that miR-125a-5p could inhibit cell glycolysis and PASMC proliferation to improve MCT-PH by targeting HK-II, which provides new insights and an understanding of the pathogenesis and a potential method of targeted therapy for MCT-PH.

## MATERIALS AND METHODS

### Establishment of a PH rat animal model

All animal experimental procedures were performed in accordance with relevant guidelines and regulations and approved by the Institutional Animal Care and Use Committee of Fujian Medical University

A PH model was successfully established through intraperitoneal injection of monocrotaline (MCT) (MCT; Sigma-Aldrich, St. Louis, MO) into rats during the early stages of the research study [7]. Eighteen SD rats (Slac Laboratory Animals Co. Ltd) were randomly divided into 3 groups: normal control (Ctr), monocrotaline-induced pulmonary arterial hypertension (MCT-PH)+NC and MCT-PH+ agomiR-125a. Rats in the MCT-PH+NC and MCT-PH+agomiR-125a groups were intraperitoneally administered 40 mg/kg MCT, while those in the Ctr group were injected with an equal volume of saline. Two weeks later, miR-125a agomir (50nM; Huzhou Hippo Biotechnology Co., Ltd, China) or normal saline (0.9% NaCl) were injected every 4 days to rats and subjected to MCT until 5 weeks.

### Nuclear magnetic resonance (NMR) spectrum processing and pattern recognition analysis

The serum metabolites were measured in each group 5 weeks after the model was established. Serum samples were collected from the abdominal aorta of the rats, and NMR spectra were mechanically obtained. Each serum acquisition parameter and its specific setting were set as follows: Temperature (K): 298.15, Magnet Frequency (MHz): 599.83, Transients/Scans: 64, Frequency Domain Size: 65536, Spectral Width: 7225.34, Time Domain Size: 28902 and Pulse Sequence: metnoesy.

The absolute concentration of the metabolites was obtained through formula conversion using known DSS internal standard concentrations and its peak area was combined with peak areas of different metabolites on the NMR spectrum. The variable matrix was used as source data for subsequent PCA and PLS-DA analyses. First, principal component analysis (PCA) was conducted to distinguish between different groups of metabolic patterns, followed by partial least squares discriminant analysis (PLS-DA). PCA and PLS-DA were performed by conducting a data analysis using the PCA methods Bioconductor package and PLS package of R language, respectively. Finally a visual map was constructed using the ggplot2 package.

### Western blotting analysis

Whole cell lysates from the cells and pulmonary arterioles were prepared in a lysis buffer (Solarbio, China) with protease/phosphatase inhibitor cocktail (Cell Signaling Technology, USA). Then, the concentration of the extracted protein was determined using a BCA protein assay kit (Solarbio, China). After equal amounts of total proteins (20 ug) were separated using SDS-PAGE and then transferred onto a nitrocellulose membrane, the membrane was incubated with primary antibodies against β-actin (8H10D10, CST, USA), HK-II (2106, CST, USA) and proliferating cell nuclear antigen (PCNA) (13110, CST, USA), at a dilution of 1:2500, followed by horseradish peroxidase-conjugated goat anti-rabbit or anti-mouse secondary antibodies (Sigma, USA), at a dilution of 1:2500. Finally, the protein bands were visualized using an enhanced chemiluminescence substrate (Western Lightning VR Plus-ECL), and the relative abundances of the target proteins were measured using ImageQuant TL software. β-actin (CST, 1:2000) served as the internal reference in this study.

### Isolation and culture of primary cells of PASMCs

Pulmonary arteries were rapidly obtained onto an ultra-clean platform. The pulmonary arteries obtained were placed in a digestive solution containing 5 mg/ml type I collagenase, 1 mg/ml papain and 1 mg/ml BSA and were digested at 37°C for 20 minutes. Each digested pulmonary artery was removed from the digestive solution using a nylon mesh and rinsed with D-Hanks solution 2-3 times. Then, the digested pulmonary artery was transferred into a small beaker with 1 ml of D-Hanks solution and pipetted slowly to disperse the cells. The cell suspension was transferred into a culture flask and was thereafter added into a DMEM low-sugar medium containing 15% fetal bovine serum and cultured at 37°C in a 5% CO_2_ incubator, and cell fusion reached 70-80% after about one week. The PASMCs were isolated from the control and the MCT-PH rats and primary cultured PASMCs were used in all experiments.

### Construction of the HK-II overexpressing lentivirus and gene chip detection

The PASMCs were transfected *in vitro* and then used to screen for miRNA molecules that were negatively correlated with HK-II expression. The control group and PASMCs overexpressing HK-II were detected using miRNA gene expression profiling.

The TRIzol method was used to extract and purify total RNA from the PASMC samples and was tested using NanoDrop 2000 and Agilent Bioanalyzer 2100. The extracted total RNA was obtained as double-stranded cDNA from the RNA, and a FlashTag Biotin HSR RNA Labeling Kit reagent was used to connect fluorescent dyes, Cy5 and Cy3, to dUTP. Fragmented cRNA was eluted and stained on a GeneChip® Fluidics Station 450, in a GeneChip® Hybridization Oven 645. A GeneChip® Scanner 3000 7G was used to scan the hybridized chips and the data was extracted to be used in calculations.

### microRNA target gene prediction

Differentially expressed microRNAs were screened using MIRANDA to predict genes regulated by these differentially expressed microRNAs, and DAVID (https://david.ncifcrf.gov/) was used to perform Gene Ontology analysis of molecular functions and biological processes on the proteins transcribed by the genes predicted.

### Real-time quantitative polymerase (RT-qPCR)

The primers were designed based on the predicted microRNA maturation sequences. Primer information was used to verify the expression of the microRNAs in the primary PASMCs of MCT-PH rats. Normal control cells and MCT were transfected with microRNA agomir and transfected with a mimic negative control (NC). MicroRNA was extracted using QIAGEN's miRNeasy® Mini Kit, while RNA concentration and purity were detected using a ultra-micro UV-visible spectrophotometer.

Total RNA was extracted from the PASMCs using TRIzol reagent (Invitrogen, USA), as instructed by the manufacturer and treated using DNase I. Then 2 ug of total RNA was reverse-transcribed using random primers and a Reverse Transcription Kit (Promega, USA). The first-strand cDNA samples obtained were then torrent-amplified using SYBRVR Green I on a Rotor-Gene 3000 A (Corbett Robotics, Australia). All PCR cycling conditions were modified to 94°C for 2 min, followed by 40 cycles of 95°C for 15s, 60°C for 15s, and 72°C for 30s. The relative changes in mRNA expression were calculated using the 2-ΔΔCt method, which was normalized using β-actin. The experiment was repeated thrice on each group. The primers were designed based on mRNA sequences and were synthesized by Shanghai Generay Biotech Co., Ltd. (Table 1).

### Verification of the relationship between microRNAs and HK-II gene targeting

The predicted wild-type (wt) miR-125a binding sequences of the 3’-UTR HK-II were synthesized and cloned downstream of the luciferase gene in the pmirGLO luciferase vector (Promega) to generate HK-II-wt. Their corresponding mutated sequences (mut) were created using a GeneTailor Site-Directed Mutagenesis System (Invitrogen) and were cloned into the same vector and named as HK-II-mut. Prior to transfection, 293T cells were seeded into 24-well plates (1 × 103 cells/well) and cultured for 24 h. Then, the 293T cells were transiently cotransfected with 100 ng of wt or mut of HK-II 3’-UTR and 10 nM of miR-125a or miR-NC, together with 20 ng of Renilla luciferase vector (Promega) using Lipofectamine 2000. The cells were collected 48 h post-transfection and their luciferase activity was measured through a luciferase reporter assay conducted using a Dual-Luciferase reporter assay system (Promega). Firefly luciferase activity was normalized to Renilla luciferase activity.

### microRNA cell transfection

The microRNA agomir and mimics agomir NC were diluted with 250 μL of Opti-MEM, and 10 μL of the transfection reagent was diluted with 250 μL of Opti-MEM, then the two diluted solutions were mixed and left to stand for 15 min. The medium in the 35 mm culture dish was discarded and 1000 μL of fresh medium was added to the above mentioned 500 μL mixture. After transfection for 24 hours, the culture solution was discarded, washed once with PBS and replaced with 1500 μL of fresh culture solution.

miR-125a agomir design: NC control: sense, 5'-UUCUCCGAACGUGUCACGUTT- 3', antisense, 5'-ACGUGACACGUUCGGAGAATT-3'; miR-125a agomir: sense, 5'- UCCCUGAGACCCUUUAACCUGUGA-3', antisense, 5'-ACAGGUUAAAGGGUCUCAGGGAUU-3'.

### CCK-8 method for detecting cell proliferation

CCK-8 solution (10 μL) was added into the 96-wells plates, then the plates were incubated for 0.5 to 4 hours and OD at 450 nm was measured using a microplate reader. [(MCT OD value - blank OD value) - (Control OD value - blank OD value)] / Control OD value.

### Detection of miRNA-125a-5p expression

The expression level of miRNA-125a-5p was measured using an Applied Biosystems miR-125 family detection kit (TaqMan MicroRNA Assays).

### Detection of glucose consumption and lactic acid content

The ketone-sulfuric acid colorimetric method and lactic acid detection kit (Solarbio) were used to measure glucose consumption and lactic acid production, respectively, as a reflection of glycolysis.

### Measurement of mPAP and right ventricular hypertrophy index (RVHI)

After treatment, the rats were anesthetized using 10% chloral hydrate (400 mg/kg). In brief, polyethylene micro-catheters (Chinese Peking Union Medical Physiology) were inserted into the pulmonary artery via the right external jugular vein and connected to a transducer. The mPAP data were collected and analyzed using Powerlab-ML221 (AD Instruments, New South Wales, Australia). RVHI was assessed by weighing the RV separately from the left ventricle (LV) with the septal wall (SW).

### Pulmonary vascular remodeling analysis

Paraffin-embedded sections were prepared and stained with hematoxylin and eosin (H&E). Pulmonary arterioles with an external diameter (ED) of 50-200 μm were chosen for the morphological analysis. Wall thickness (WT) and ED of the pulmonary arteries were measured using IPP 6.0 image analysis software (Media Cybernetics). The remodeling of the pulmonary arterioles were calculated as WT% = 2 x WT/ED x100%.

### Cardiac ultrasound detection

Color Doppler Ultrasound Diagnostic (GE gray-E9), with a probe frequency of 12 MHz, which maintains a frame rate of 180-220 s when acquiring images was used. SD rats were found to have a fast heart rate of about 400 beats per minute. Ultrasound measurement parameters were based on standards established by the American Society of Echocardiography [19], along with a setting of Doppler flow spectrum and a M-curve scan speed of > l00 mm/s. The SD rats were anesthetized using pentobarbital injection (120 mg/kg, intraperitoneal injection), placed on an examination bed and the coupling agent was applied to left chest.

During the ultrasonography, the II lead electrocardiogram was recorded synchronously and heart rate was measured. The apex of the R wave was the diastole end, and the end of the T wave was the systole end. Pulmonary artery acceleration time (PAAT) was measured by placing probe next to sternum. A M-mode ultrasound was used to measure right ventricular end thickness (RVWT), right ventricular end systolic diameter (RVESD), right ventricular end diastolic diameter (RVEED) and tricuspid annular systolic excursion (TAPSE) in the apical four-chamber view. The amplitude was measured from the end-diastolic phase to the end-systolic phase. In general, data was measured in 3 cardiac cycles and averaged.

### Statistical analysis

PCA and PLS-DA statistical analyses were performed on NMR data using Chenomx NMR Suite 8.0 software. The quality of the PLS-DA model was evaluated using a permutation test. The SPSS 23.0 statistical software package was used to analyze specific metabolite concentration data. All measured data are presented as mean ±standard deviation. Statistical analysis for multiple group comparisons were performed by one-way analysis of variance (ANOVA), followed by post-hoc Dunnett’s test. A p value of less than 0.05 was considered to be significant. All experiments described above were performed at least three times.

## References

[1] Working Group on Pulmonary Vascular Diseases of Chinese Society of Cardiology of Chinese Medical Association; Editorial Board of Chinese Journal of Cardiology. [Chinese guidelines for diagnosis and treatment of pulmonary hypertension 2018.] Zhonghua Xin Xue Guan Bing Za Zhi. 2018; 46:933–64. 10.3760/cma.j.issn.0253-3758.2018.12.006 30572400

[2] Jing ZC, Xu XQ, Han ZY, Wu Y, Deng KW, Wang H, Wang ZW, Cheng XS, Xu B, Hu SS, Hui RT, Yang YJ. Registry and survival study in Chinese patients with idiopathic and familial pulmonary arterial hypertension. Chest. 2007; 132:373–79. 10.1378/chest.06-2913 17400671

[3] Zhang R, Dai LZ, Xie WP, Yu ZX, Wu BX, Pan L, Yuan P, Jiang X, He J, Humbert M, Jing ZC. Survival of chinese patients with pulmonary arterial hypertension in the modern treatment era. Chest. 2011; 140:301–09. 10.1378/chest.10-2327 21330386

[4] Warburg O. On respiratory impairment in cancer cells. Science. 1956; 124:269–70. 13351639

[5] Jiang SH, Li J, Dong FY, Yang JY, Liu DJ, Yang XM, Wang YH, Yang MW, Fu XL, Zhang XX, Li Q, Pang XF, Huo YM, et al. Increased serotonin signaling contributes to the warburg effect in pancreatic tumor cells under metabolic stress and promotes growth of pancreatic tumors in mice. Gastroenterology. 2017; 153:277–291.e19. 10.1053/j.gastro.2017.03.008 28315323

[6] Marsboom G, Wietholt C, Haney CR, Toth PT, Ryan JJ, Morrow E, Thenappan T, Bache-Wiig P, Piao L, Paul J, Chen CT, Archer SL. Lung ¹^8^F-fluorodeoxyglucose positron emission tomography for diagnosis and monitoring of pulmonary arterial hypertension. Am J Respir Crit Care Med. 2012; 185:670–79. 10.1164/rccm.201108-1562OC 22246173 PMC3326289

[7] Luo L, Zheng W, Lian G, Chen H, Li L, Xu C, Xie L. Combination treatment of adipose-derived stem cells and adiponectin attenuates pulmonary arterial hypertension in rats by inhibiting pulmonary arterial smooth muscle cell proliferation and regulating the AMPK/BMP/smad pathway. Int J Mol Med. 2018; 41:51–60. 10.3892/ijmm.2017.3226 29115380 PMC5746303

[8] Lu Z, Guo Y, Zhang X, Li J, Li L, Zhang S, Shan C. ORY-1001 suppresses cell growth and induces apoptosis in lung cancer through triggering HK2 mediated warburg effect. Front Pharmacol. 2018; 9:1411. 10.3389/fphar.2018.01411 30568590 PMC6290890

[9] Mathupala SP, Rempel A, Pedersen PL. Glucose catabolism in cancer cells. Isolation, sequence, and activity of the promoter for type II hexokinase. J Biol Chem. 1995; 270:16918–25. 10.1074/jbc.270.28.16918 7622509

[10] Mathupala SP, Rempel A, Pedersen PL. Glucose catabolism in cancer cells: identification and characterization of a marked activation response of the type II hexokinase gene to hypoxic conditions. J Biol Chem. 2001; 276:43407–12. 10.1074/jbc.M108181200 11557773

[11] Song J, Wu X, Liu F, Li M, Sun Y, Wang Y, Wang C, Zhu K, Jia X, Wang B, Ma X. Long non-coding RNA PVT1 promotes glycolysis and tumor progression by regulating miR-497/HK2 axis in osteosarcoma. Biochem Biophys Res Commun. 2017; 490:217–24. 10.1016/j.bbrc.2017.06.024 28602700

[12] Londin E, Loher P, Telonis AG, Quann K, Clark P, Jing Y, Hatzimichael E, Kirino Y, Honda S, Lally M, Ramratnam B, Comstock CE, Knudsen KE, et al. Analysis of 13 cell types reveals evidence for the expression of numerous novel primate- and tissue-specific microRNAs. Proc Natl Acad Sci USA. 2015; 112:E1106–15. 10.1073/pnas.1420955112 25713380 PMC4364231

[13] Fang R, Xiao T, Fang Z, Sun Y, Li F, Gao Y, Feng Y, Li L, Wang Y, Liu X, Chen H, Liu XY, Ji H. MicroRNA-143 (miR-143) regulates cancer glycolysis via targeting hexokinase 2 gene. J Biol Chem. 2012; 287:23227–35. 10.1074/jbc.M112.373084 22593586 PMC3391126

[14] Wei J, Ma Z, Li Y, Zhao B, Wang D, Jin Y, Jin Y. miR-143 inhibits cell proliferation by targeting autophagy-related 2B in non-small cell lung cancer H1299 cells. Mol Med Rep. 2015; 11:571–76. 10.3892/mmr.2014.2675 25322940

[15] Jiang S, Zhang LF, Zhang HW, Hu S, Lu MH, Liang S, Li B, Li Y, Li D, Wang ED, Liu MF. A novel miR-155/miR-143 cascade controls glycolysis by regulating hexokinase 2 in breast cancer cells. EMBO J. 2012; 31:1985–98. 10.1038/emboj.2012.45 22354042 PMC3343331

[16] Lin TJ. Mechanisms and effects of atorvastatin on monocrotaline-induced pulmonary arterial hypertension in rats by 1NMR-based metabonomic analysis. Fujian Medicine University 2013.

[17] Montani D, Günther S, Dorfmüller P, Perros F, Girerd B, Garcia G, Jaïs X, Savale L, Artaud-Macari E, Price LC, Humbert M, Simonneau G, Sitbon O. Pulmonary arterial hypertension. Orphanet J Rare Dis. 2013; 8:97. 10.1186/1750-1172-8-97 23829793 PMC3750932

[18] Tuder RM, Marecki JC, Richter A, Fijalkowska I, Flores S. Pathology of pulmonary hypertension. Clin Chest Med. 2007; 28:23–42. 10.1016/j.ccm.2006.11.010 17338926 PMC1924722

[19] Dai J, Zhou Q, Chen J, Rexius-Hall ML, Rehman J, Zhou G. Alpha-enolase regulates the Malignant phenotype of pulmonary artery smooth muscle cells via the AMPK-Akt pathway. Nat Commun. 2018; 9:3850. 10.1038/s41467-018-06376-x 30242159 PMC6155017

[20] Wakasugi T, Shimizu I, Yoshida Y, Hayashi Y, Ikegami R, Suda M, Katsuumi G, Nakao M, Hoyano M, Kashimura T, Nakamura K, Ito H, Nojiri T, et al. Role of smooth muscle cell p53 in pulmonary arterial hypertension. PLoS One. 2019; 14:e0212889. 10.1371/journal.pone.0212889 30807606 PMC6391010

[21] Vander Heiden MG, Cantley LC, Thompson CB. Understanding the warburg effect: the metabolic requirements of cell proliferation. Science. 2009; 324:1029–33. 10.1126/science.1160809 19460998 PMC2849637

[22] Li W, Gao F, Ma X, Wang R, Dong X, Wang W. Deguelin inhibits non-small cell lung cancer via down-regulating hexokinases II-mediated glycolysis. Oncotarget. 2017; 8:32586–99. 10.18632/oncotarget.15937 28427230 PMC5464811

[23] Takahashi M, Makino S, Kikkawa T, Osumi N. Preparation of rat serum suitable for mamMalian whole embryo culture. J Vis Exp. 2014; 90:e51969. 10.3791/51969 25145996 PMC4672955

[24] Thenappan T, Ormiston ML, Ryan JJ, Archer SL. Pulmonary arterial hypertension: pathogenesis and clinical management. BMJ. 2018; 360:j5492. 10.1136/bmj.j5492 29540357 PMC6889979

[25] Moudgil R, Michelakis ED, Archer SL. The role of K+ channels in determining pulmonary vascular tone, oxygen sensing, cell proliferation, and apoptosis: implications in hypoxic pulmonary vasoconstriction and pulmonary arterial hypertension. Microcirculation. 2006; 13:615–32. 10.1080/10739680600930222 17085423

[26] Malczyk M, Veith C, Fuchs B, Hofmann K, Storch U, Schermuly RT, Witzenrath M, Ahlbrecht K, Fecher-Trost C, Flockerzi V, Ghofrani HA, Grimminger F, Seeger W, et al. Classical transient receptor potential channel 1 in hypoxia-induced pulmonary hypertension. Am J Respir Crit Care Med. 2013; 188:1451–59. 10.1164/rccm.201307-1252OC 24251695

[27] Nogueira-Ferreira R, Vitorino R, Ferreira R, Henriques-Coelho T. Exploring the monocrotaline animal model for the study of pulmonary arterial hypertension: a network approach. Pulm Pharmacol Ther. 2015; 35:8–16. 10.1016/j.pupt.2015.09.007 26403584

[28] Zhang WH, Qiu MH, Wang XJ, Sun K, Zheng Y, Jing ZC. Up-regulation of hexokinase1 in the right ventricle of monocrotaline induced pulmonary hypertension. Respir Res. 2014; 15:119. 10.1186/s12931-014-0119-9 25287584 PMC4198683

[29] Zhang C, Ma C, Zhang L, Zhang L, Zhang F, Ma M, Zheng X, Mao M, Shen T, Zhu D. MiR-449a-5p mediates mitochondrial dysfunction and phenotypic transition by targeting Myc in pulmonary arterial smooth muscle cells. J Mol Med (Berl). 2019; 97:409–22. 10.1007/s00109-019-01751-7 30715622

[30] Aliotta JM, Pereira M, Wen S, Dooner MS, Del Tatto M, Papa E, Goldberg LR, Baird GL, Ventetuolo CE, Quesenberry PJ, Klinger JR. Exosomes induce and reverse monocrotaline-induced pulmonary hypertension in mice. Cardiovasc Res. 2016; 110:319–30. 10.1093/cvr/cvw054 26980205 PMC4872877

[31] Guo W, Qiu Z, Wang Z, Wang Q, Tan N, Chen T, Chen Z, Huang S, Gu J, Li J, Yao M, Zhao Y, He X. MiR-199a-5p is negatively associated with malignancies and regulates glycolysis and lactate production by targeting hexokinase 2 in liver cancer. Hepatology. 2015; 62:1132–44. 10.1002/hep.27929 26054020

[32] Fagerberg L, Hallström BM, Oksvold P, Kampf C, Djureinovic D, Odeberg J, Habuka M, Tahmasebpoor S, Danielsson A, Edlund K, Asplund A, Sjöstedt E, Lundberg E, et al. Analysis of the human tissue-specific expression by genome-wide integration of transcriptomics and antibody-based proteomics. Mol Cell Proteomics. 2014; 13:397–406. 10.1074/mcp.M113.035600 24309898 PMC3916642

[33] Lin T, Gu J, Huang C, Zheng S, Lin X, Xie L, Lin D. (1)H NMR-based analysis of serum metabolites in monocrotaline-induced pulmonary arterial hypertensive rats. Dis Markers. 2016; 2016:5803031. 10.1155/2016/5803031 27057080 PMC4745193

[34] Xu D, Jin J, Yu H, Zhao Z, Ma D, Zhang C, Jiang H. Chrysin inhibited tumor glycolysis and induced apoptosis in hepatocellular carcinoma by targeting hexokinase-2. J Exp Clin Cancer Res. 2017; 36:44. 10.1186/s13046-017-0514-4 28320429 PMC5359903

[35] Pastorino JG, Hoek JB, Shulga N. Activation of glycogen synthase kinase 3beta disrupts the binding of hexokinase II to mitochondria by phosphorylating voltage-dependent anion channel and potentiates chemotherapy-induced cytotoxicity. Cancer Res. 2005; 65:10545–54. 10.1158/0008-5472.CAN-05-1925 16288047

[36] Roberts DJ, Tan-Sah VP, Ding EY, Smith JM, Miyamoto S. hexokinase-II positively regulates glucose starvation-induced autophagy through TORC1 inhibition. Mol Cell. 2014; 53:521–33. 10.1016/j.molcel.2013.12.019 24462113 PMC3943874

[37] Lee HG, Kim H, Son T, Jeong Y, Kim SU, Dong SM, Park YN, Lee JD, Lee JM, Park JH. Regulation of HK2 expression through alterations in CpG methylation of the HK2 promoter during progression of hepatocellular carcinoma. Oncotarget. 2016; 7:41798–810. 10.18632/oncotarget.9723 27260001 PMC5173097

[38] Caruso P, Dunmore BJ, Schlosser K, Schoors S, Dos Santos C, Perez-Iratxeta C, Lavoie JR, Zhang H, Long L, Flockton AR, Frid MG, Upton PD, D'Alessandro A, et al. Identification of MicroRNA-124 as a Major Regulator of Enhanced Endothelial Cell Glycolysis in Pulmonary Arterial Hypertension via PTBP1 (Polypyrimidine Tract Binding Protein) and Pyruvate Kinase M2. Circulation. 2017; 136:2451–67. 10.1161/CIRCULATIONAHA.117.028034 28971999 PMC5736425

[39] Zhang H, Wang D, Li M, Plecitá-Hlavatá L, D’Alessandro A, Tauber J, Riddle S, Kumar S, Flockton A, McKeon BA, Frid MG, Reisz JA, Caruso P, et al. Metabolic and proliferative state of vascular adventitial fibroblasts in pulmonary hypertension is regulated through a MicroRNA-124/PTBP1 (Polypyrimidine tract binding protein 1)/pyruvate kinase muscle axis. Circulation. 2017; 136:2468–85. 10.1161/CIRCULATIONAHA.117.028069 28972001 PMC5973494

[40] Hong Z, Chen KH, DasGupta A, Potus F, Dunham-Snary K, Bonnet S, Tian L, Fu J, Breuils-Bonnet S, Provencher S, Wu D, Mewburn J, Ormiston ML, Archer SL. MicroRNA-138 and MicroRNA-25 down-regulate mitochondrial calcium uniporter, causing the pulmonary arterial hypertension cancer phenotype. Am J Respir Crit Care Med. 2017; 195:515–29. 10.1164/rccm.201604-0814OC 27648837 PMC5378421

[41] Kim J, Kang Y, Kojima Y, Lighthouse JK, Hu X, Aldred MA, McLean DL, Park H, Comhair SA, Greif DM, Erzurum SC, Chun HJ. An endothelial apelin-FGF link mediated by miR-424 and miR-503 is disrupted in pulmonary arterial hypertension. Nat Med. 2013; 19:74–82. 10.1038/nm.3040 23263626 PMC3540168

[42] Brock M, Samillan VJ, Trenkmann M, Schwarzwald C, Ulrich S, Gay RE, Gassmann M, Ostergaard L, Gay S, Speich R, Huber LC. AntagomiR directed against miR-20a restores functional BMPR2 signalling and prevents vascular remodelling in hypoxia-induced pulmonary hypertension. Eur Heart J. 2014; 35:3203–11. 10.1093/eurheartj/ehs060 22450430

[43] Kriegel AJ, Baker MA, Liu Y, Liu P, Cowley AW Jr, Liang M. Endogenous microRNAs in human microvascular endothelial cells regulate mRNAs encoded by hypertension-related genes. Hypertension. 2015; 66:793–99. 10.1161/HYPERTENSIONAHA.115.05645 26283043 PMC4567475

[44] Cai Z, Li J, Zhuang Q, Zhang X, Yuan A, Shen L, Kang K, Qu B, Tang Y, Pu J, Gou D, Shen J. MiR-125a-5p ameliorates monocrotaline-induced pulmonary arterial hypertension by targeting the TGF-β1 and IL-6/STAT3 signaling pathways. Exp Mol Med. 2018; 50:45. 10.1038/s12276-018-0068-3 29700287 PMC5938047

[45] Li D, Yang P, Xiong Q, Song X, Yang X, Liu L, Yuan W, Rui YC. MicroRNA-125a/b-5p inhibits endothelin-1 expression in vascular endothelial cells. J Hypertens. 2010; 28:1646–54. 10.1097/HJH.0b013e32833a4922 20531225

[46] Jia CW, Sun Y, Zhang TT, Lu ZH, Chen J. Effects of miR-125a-5p on cell Proliferation, apoptosis and cell cycle of pancreatic cancer cells. Zhongguo Yi Xue Ke Xue Yuan Xue Bao. 2016; 38:415–21. 27594154 10.3881/j.issn.1000-503X.2016.04.009

[47] Liu J, Tang Q, Li S, Yang X. Inhibition of HAX-1 by miR-125a reverses cisplatin resistance in laryngeal cancer stem cells. Oncotarget. 2016; 7:86446–56. 10.18632/oncotarget.13424 27880721 PMC5349925

[48] Yin H, Sun Y, Wang X, Park J, Zhang Y, Li M, Yin J, Liu Q, Wei M. Progress on the relationship between miR-125 family and tumorigenesis. Exp Cell Res. 2015; 339:252–60. 10.1016/j.yexcr.2015.09.015 26407906

[49] Natalia MA, Alejandro GT, Virginia TJ, Alvarez-Salas LM. MARK1 is a novel target for miR-125a-5p: implications for cell migration in cervical tumor cells. Microrna. 2018; 7:54–61. 10.2174/2211536606666171024160244 29076440

[50] Cao Y, Tan S, Tu Y, Zhang G, Liu Y, Li D, Xu S, Le Z, Xiong J, Zou W, Gong P, Li Z, Jie Z. MicroRNA-125a-5p inhibits invasion and metastasis of gastric cancer cells by targeting BRMS1 expression. Oncol Lett. 2018; 15:5119–30. 10.3892/ol.2018.7983 29552146 PMC5840665

[51] Tong Z, Liu N, Lin L, Guo X, Yang D, Zhang Q. miR-125a-5p inhibits cell proliferation and induces apoptosis in colon cancer via targeting BCL2, BCL2L12 and MCL1. Biomed Pharmacother. 2015; 75:129–36. 10.1016/j.biopha.2015.07.036 26297542

[52] Li W, Qiu Y, Hao J, Zhao C, Deng X, Shu G. Dauricine upregulates the chemosensitivity of hepatocellular carcinoma cells: role of repressing glycolysis via miR-199a:HK2/PKM2 modulation. Food Chem Toxicol. 2018; 121:156–65. 10.1016/j.fct.2018.08.030 30171973

[53] Xia F, Sun JJ, Jiang YQ, Li CF. MicroRNA-384-3p inhibits retinal neovascularization through targeting hexokinase 2 in mice with diabetic retinopathy. J Cell Physiol. 2018; 234:721–30. 10.1002/jcp.26871 30191948

[54] Zhang K, Zhang M, Jiang H, Liu F, Liu H, Li Y. Down-regulation of miR-214 inhibits proliferation and glycolysis in non-small-cell lung cancer cells via down-regulating the expression of hexokinase 2 and pyruvate kinase isozyme M2. Biomed Pharmacother. 2018; 105:545–52. 10.1016/j.biopha.2018.06.009 29886375

[55] Hui L, Zhang J, Guo X. MiR-125b-5p suppressed the glycolysis of laryngeal squamous cell carcinoma by down-regulating hexokinase-2. Biomed Pharmacother. 2018; 103:1194–201. 10.1016/j.biopha.2018.04.098 29864898

